# Cardioluminescence in Transgenic Zebrafish Larvae: A Calcium Imaging Tool to Study Drug Effects and Pathological Modeling

**DOI:** 10.3390/biomedicines9101294

**Published:** 2021-09-22

**Authors:** Manuel Vicente, Jussep Salgado-Almario, Michelle M. Collins, Antonio Martínez-Sielva, Masafumi Minoshima, Kazuya Kikuchi, Beatriz Domingo, Juan Llopis

**Affiliations:** 1 Physiology and Cell Dynamics Group, Centro Regional de Investigaciones Biomédicas (CRIB) and Facultad de Medicina de Albacete, Universidad de Castilla-La Mancha, C/Almansa 14, 02006 Albacete, Spain; manuel.vicente@uclm.es (M.V.); Jussep.Salgado@uclm.es (J.S.-A.); antonio.martinez34@alu.uclm.es (A.M.-S.); 2 Department of Anatomy, Physiology, and Pharmacology, College of Medicine, University of Saskatchewan, Saskatoon, SK S7N 5E5, Canada; michelle.collins@usask.ca; 3 Department of Material and Life Science, Graduate School of Engineering, Osaka University, Suita, Osaka 565-0871, Japan; minoshima@mls.eng.osaka-u.ac.jp (M.M.); kkikuchi@mls.eng.osaka-u.ac.jp (K.K.); 4 WPI-Immunology Frontier Research Center, Osaka University, Suita, Osaka 565-0871, Japan

**Keywords:** zebrafish, calcium, heart, aequorin, coelenterazine, heart failure, imaging

## Abstract

Zebrafish embryos and larvae have emerged as an excellent model in cardiovascular research and are amenable to live imaging with genetically encoded biosensors to study cardiac cell behaviours, including calcium dynamics. To monitor calcium ion levels in three to five days post-fertilization larvae, we have used bioluminescence. We generated a transgenic line expressing GFP-aequorin in the heart, *Tg(myl7:GA)*, and optimized a reconstitution protocol to boost aequorin luminescence. The analogue *diacetyl* *h*-coelenterazine enhanced light output and signal-to-noise ratio. With this cardioluminescence model, we imaged the time-averaged calcium levels and beat-to-beat calcium oscillations continuously for hours. As a proof-of-concept of the transgenic line, changes in ventricular calcium levels were observed by Bay K8644, an L-type calcium channel activator and with the blocker nifedipine. The β-adrenergic blocker propranolol decreased calcium levels, heart rate, stroke volume, and cardiac output, suggesting that larvae have a basal adrenergic tone. Zebrafish larvae treated with terfenadine for 24 h have been proposed as a model of heart failure. *Tg(myl7:GA)* larvae treated with terfenadine showed bradycardia, 2:1 atrioventricular block, decreased time-averaged ventricular calcium levels but increased calcium transient amplitude, and reduced cardiac output. As alterations of calcium signalling are involved in the pathogenesis of heart failure and arrhythmia, the GFP-aequorin transgenic line provides a powerful platform for understanding calcium dynamics.

## 1. Introduction

Cardiovascular diseases are the leading cause of death worldwide and are among the most challenging to diagnose and treat due to the complexity of their pathophysiology. Ca^2+^ plays a pivotal role in the excitation–contraction coupling of the heart and alterations in Ca^2+^ cycling and its associated proteins and pathways may trigger pathological disorders [1,2]. Ca^2+^ handling has been extensively studied, mainly in isolated cardiomyocytes obtained from animals or from differentiated human pluripotent stem cells [3,4,5,6]. While this approach provides a detailed insight of the Ca^2+^ fluxes, the interaction between different heart cells, other organs, and the nervous and endocrine systems is lost. Thus, to fully understand pathological processes, in vivo animal models are crucial.

The zebrafish has emerged as an excellent model in cardiovascular research because of its high genetic tractability, ex-utero embryonic development, and transparency [7,8,9,10,11,12,13]. The zebrafish heart is composed of only two chambers, the atrium and ventricle. Despite this anatomical difference, zebrafish heart physiology, heart rate (HR), and action potential resemble those of humans [14,15,16], although some of the ionic channels and regulation are not identical [17,18].

Non-invasive Ca^2+^ imaging techniques in zebrafish embryos and larvae contribute to our understanding of Ca^2+^ handling and its relationship with cardiac diseases. Genetically encoded Ca^2+^ indicators, like the single-fluorophore GCaMPs, have been successfully used to image Ca^2+^ dynamics in the embryonic heart [11,19,20,21]. They require uncoupling of contraction from Ca^2+^ changes either by using morpholino oligomers against troponin T [22] or with myosin II inhibitors like *para*-amino blebbistatin [23] to avoid motion imaging artifacts. We have previously used ratiometric biosensors, which call for special optical components to acquire two emission images simultaneously to correct these artifacts [24]. Fluorescent biosensors provide excellent temporal and spatial resolution of the cardiac Ca^2+^ transients, but suffer from some pitfalls, particularly for imaging the heart. The fast HR necessitates acquiring time-lapse images with minimal interval between them so that the dynamics of the Ca^2+^ transients may be recorded. In practice, this limits the duration of an experiment to a few seconds at a time, depending on the instrumentation, to avoid excessive photobleaching. A solution is the use of low-light-level techniques like light sheet microscopy, which is less harmful to the cells than widefield microscopy [20,25], but more costly. A second drawback is the autofluorescence of the vitello, adjacent to the heart, or of commonly used drugs like *para*-amino blebbistatin. 

These technical issues prompted us to explore aequorin bioluminescence to image heart Ca^2+^ continuously for hours, acquiring all emitted photons during that time. By contrast with fluorescence, bioluminescence does not need excitation light. The photoprotein aequorin has been widely employed to measure Ca^2+^ in many cell types and animal models [26,27,28] and has been demonstrated to be biocompatible in zebrafish [29,30,31,32]. The functional photoprotein is formed when apoaequorin binds the substrate coelenterazine (CTZ) [26]. In our previous work, aequorin fused with GFP (GA) [33] was used to visualize cytoplasmic and mitochondrial Ca^2+^ in skeletal muscle of zebrafish embryos for hours [32]. Despite its low photon yield compared with fluorescence, we hypothesized that GA could be used to measure the synchronized Ca^2+^ transients without the need to stop heart beating, thus, conserving the physiological mechano-electrical feedback mechanisms. However, we anticipated that it might be challenging to achieve sufficient reconstitution with aequorin’s substrate CTZ in the presence of constant Ca^2+^ cycling, since aequorin is consumed once it emits light.

In this study, we have generated a transgenic zebrafish line expressing GA to image continuously ventricular Ca^2+^ levels in vivo (cardioluminescence). Ca^2+^ was measured as the heart was performing its mechanical function in three-, four-, and five-days post-fertilization (dpf) larvae. To improve the efficiency of aequorin reconstitution with CTZ, a protocol was devised to decrease temporarily Ca^2+^ cycling in the heart. The zebrafish line *Tg(myl7:GA)* allowed imaging both the time-averaged levels and the systolic Ca^2+^ transients continuously for several hours in control conditions, in response to drugs, and in a model of heart failure induced by terfenadine. 

## 2. Materials and Methods

### 2.1. Zebrafish Husbandry

Fish used in this study were housed under standard conditions as previously described [32]. 

### 2.2. Generation of Tg(myl7:GA) Zebrafish Line

The Ca^2+^ biosensor GFP-aequorin (GA) [33] was cloned into the *pT2A-Tol2-myl7-MCS* transposon vector (0.8 kb *myl7* promoter), using *Xho*I and *Eco*RI restriction sites. To generate stable transgenic zebrafish, *tol2-myl7:GA* plasmid (12.5 ng/µL) was co-injected with transposase mRNA (12.5 ng/µL) in fertilized eggs of Tübingen zebrafish. Injected embryos were screened by fluorescence for GA expression in the heart and grown to adulthood (F0). The adult F0 generation was outcrossed to wild-type zebrafish to identify founders with insertions in the germline. F2 *Tg(myl7:GA)* heterozygous larvae were used throughout the study. 

### 2.3. Synthesis of Diacetyl CTZ-h

CTZ-*h* was synthesized using the procedures in a previous report [34]. *h*-CTZ (73.2 mg, 0.180 mmol) and DMAP (60.0 mg, 0.491 mmol) in acetic anhydride (2.50 mL, 26.4 mmol) were stirred overnight at 20 °C under a N_2_ atmosphere. After removal of all the volatiles, the residue was dissolved in ethyl acetate and washed with 2 M HCl, saturated aqueous NaHCO_3_, and brine, with the organic layer then dried over Na_2_SO_4_, filtered, and evaporated in vacuo. The residue was purified by silica gel column chromatography using 5–10% ethyl acetate in dichloromethane as eluent to afford *diacetyl h*-CTZ as a yellow-brown solid (64.3 mg, 0.131 mmol, 73%). 1H NMR (500 MHz, CDCl_3_): δ 7.87 (d, *J* = 9.0 Hz, 2H), 7.73 (s, 1H), 7.84 (d, *J* = 7.5 Hz, 2H), 7.30–7.20 (m, 8H), 7.15 (d, *J* = 9.0 Hz, 2H), 4.60 (s, 2H) 4.18 (s, 2H) 2.29 (s, 3H), 2.12 (s, 3H). MS (ESI+) Calcd for [M + H]^+^, 492.19; found 492.19.

### 2.4. Aequorin Reconstitution with Coelenterazine

A stock of *diacetyl h*-CTZ was prepared at 7.4 mM in dimethyl sulfoxide (DMSO). The stocks of *native*-, *hcp-, fcp*-, *h*-, and *f*-CTZ analogues (Biotium, Fremont, CA, USA) were prepared at 5 mM in methanol. All CTZ were stored at −80 °C in 5 µL aliquots and were used at 50 µM final concentration. For aequorin reconstitution, *Tg(myl7:GA*) zebrafish larvae at 3, 4, and 5 dpf were rinsed in nominally zero Ca^2+^ E3 (E3_0Ca_) medium (E3_0Ca_ medium: 5 mM NaCl, 0.17 mM KCl, 0.33 mM MgSO_4_, 0.002% methylene blue, pH 7.4 in double distilled H_2_O). Subsequently, larvae were incubated in 1 mL of E3_0Ca_ medium containing 25 µM nifedipine for 30 min at room temperature in the dark (*nifedipine period*). Larvae whose heart was completely stopped after this treatment were washed out five times to remove nifedipine and incubated in E3_0Ca_ medium containing 50 µM CTZ for 2 h at room temperature in the dark (*CTZ incubation period*). Finally, the larvae were incubated in complete E3 medium (5 mM NaCl, 0.17 mM KCl, 0.33 mM MgSO_4_, 0.33 mM CaCl_2_, 0.002% methylene blue, pH 7.4 in double distilled H_2_O) for 30 min at 28.5 °C (*heart recovery period*). 

### 2.5. Bioluminescence Imaging

After aequorin reconstitution, *Tg(myl7:GA)* larvae were embedded in 100 µL of 0.3% low melting point agarose prepared in E3 medium and transferred to an 8-well glass bottom plate (ibidi, Gräfelfing, Germany). When the agarose solidified, 100 µL of E3 medium was added and a small portion of agarose surrounding the heart was cut out to improve diffusion of drugs or Triton X-100. Bioluminescence images were acquired as previously described [29] with a custom-built microscope (Low Light Microscope [35]) equipped with an EM-CCD camera (512 × 512 pixels, EMC9100-13, Hamamatsu Photonics, Hamamatsu, Japan). All microscope components were acquired from Thorlabs GmbH (Dachau, Germany). A 4× CFI PlanApo Lambda air objective (Nikon, Tokyo, Japan) was used as the tube lens, and an air 20× Nikon CFI PlanApo Lambda (0.75 NA) as the objective. The magnification of these combined lenses (f tube lense/f objective lens) was 5×. This microscope was equipped with a LED lamp for transmitted light. The microscope was housed in a light-tight box to maintain complete darkness during bioluminescence imaging. The larvae were maintained from 26 to 28 °C during imaging. The bioluminescence images were acquired continuously in 16 bits with 4×4 binning, 255 EM gain, at a rate of 25, 17, 12, 9, 2, or 1 Hz (frames/s). With this configuration, the resolution of the images was 12.8 µm × 12.8 µm/pixel. For the signal-to-noise ratio (SNR) calculation, bioluminescence images were acquired at 9 Hz for 1 min. For longer recordings, drugs were added after 1–10 min basal tracking of luminescence. At the end of every recording, Triton X-100 (5%) was added to break embryo membranes. The released aequorin, in contact with extracellular Ca^2+^, emits all remaining luminescence counts.

### 2.6. GCaMP Fluorescence Imaging

*Tg(myl7:GCaMP)^s878^* adult zebrafish were outcrossed to wild-type strain and fertilized eggs at 1-cell stage were injected with 2 ng of the morpholino oligomer *tnnt2a* (5′-CATGTTTGCTCTGATCTGACACGCA-3′). Larvae at 24 h post-fertilization were placed in 0.003% N-phenylthiourea to prevent pigmentation. For nifedipine titration, 3 dpf larvae were incubated in E3 or E3_0Ca_ medium containing 10, 25, or 100 µM nifedipine for 30 min. Then, larvae were embedded in 1% low melting point agarose and transferred to an 8-well glass bottom plate (ibidi, Gräfeling, Germany). To study the recovery of Ca^2+^ dynamics with GCaMP, the aequorin reconstitution protocol described above was applied to 3 dpf larvae, without addition of CTZ. Fluorescence images were acquired at a rate of 200 Hz with a CSU X1 spinning disc confocal microscope (Carl Zeiss, Oberkochen, Germany) equipped with a Hamamatsu ORCA Flash4.0 sCMOS camera (Hamamatsu Photonics, Japan) in 16 bits with 2×2 binning. 

### 2.7. Reagents

Chemical compounds were dissolved in DMSO to prepare stocks of 10 mM nifedipine (Sigma-Aldrich N7634, Darmstadt, Germany), 20 mM Bay K8644 (Tocris 1544), 10 mM propranolol (Sigma-Aldrich P0884, Darmstadt, Germany), 50 mM terfenadine (Tocris 3948), and 7.5% N-phenylthiourea (Sigma-Aldrich, Darmstadt, Germany).

### 2.8. Heart Failure Induced by Treatment with Terfenadine

Embryos at 24 h post-fertilization were placed in 0.003% N-phenylthiourea in E3 medium to prevent pigmentation. At 3 dpf, larvae were transferred to a 6-well plate, 10 larvae per well, with 5 mL of N-phenylthiourea solution containing 10 µM terfenadine or 0.02% DMSO, for 24 h. For bioluminescence experiments, aequorin reconstitution was done during the last 3 h of terfenadine or DMSO treatment.

### 2.9. Data Analysis

Videos in TIFF format were analysed in Fiji-ImageJ (U.S. National Institutes of Health, Bethesda, MD, USA) [36]. For GCaMP image analysis, regions of interest (ROI) were drawn in the atrium and in the ventricle to obtain mean intensity values. An exponentially weighted moving average smoothing with a factor of 0.7 was applied and data was transformed into ΔF/F_0_:

ΔF/F_0_ = (F_t_ − F_0_)/F_0_,

where F_t_ is the fluorescence at a given time and F_0_ is the minimum diastolic fluorescence value of the whole recording. For characterization of the Ca^2+^ transients, ΔF/F_0_ data were analysed with Clampfit 10.7 (Molecular Devices, San José, CA, USA) to determine peak amplitude ((F_systole_ − F_0_)/F_0_), rise time 10% to 90%, and decay time 90% to 10% (Figure S2D).

For bioluminescence image analysis and calculation of SNR, a time-projection of the image stack was performed to draw the ROIs over the ventricle and atrium (*Signal*). Then, ROIs of identical size were placed in six different locations far from the larva to obtain average background intensity values (*Background*) and their SD. The SNR for each frame was calculated as:

SNR = (Signal − Background)/SD Background.

Luminescence *Signal* (in relative light units, RLU) was transformed into *Luminescence rate* (*L*, in counts per second). The *Total counts* (*Ltotal*, in RLU) were obtained as the sum of luminescence along the experiment. *Lconsumed*, the sum of all *L* values from time zero to any given time point, was calculated. It represents the aequorin that has already been spent in each time point. Finally, *Lmax* was calculated as *Ltotal* − *Lconsumed* at each time point. *Lmax* represents the aequorin available at each time point: the sum of counts from that time point to the end of the experiment.

The hemodynamic parameters were analysed in Fiji-ImageJ by measuring the major and minor diameters (D), and the area (A) on the ventricular cavity at end-systole and end-diastole in three transmitted light or fluorescence images in each larva, as indicated. The major and minor diameters were measured in the longitudinal and transverse directions of the ventricle, respectively. Images were acquired at 50 Hz in a wide-field fluorescence microscope (DMIRE-2, Leica Microsystems, Wetzlar, Germany). Ventricular fractional shortening (FS) was calculated from major or minor end-diastolic (D_diastole_) and end-systolic (D_systole_) diameters, as:

FS = (D_diastole_ − D_systole_)/D_diastole_.

Fractional area change was calculated as:

FAC = (A_diastole_ − A_systole_)/A_diastole_.

End-systolic volume (ESV) and end-diastolic volume (EDV) were calculated as the volume of an ellipsoid of revolution, as reported [37]:

Volume = (π/6) × (major diameter) × (minor diameter)^2^

The stroke volume and cardiac output were:

Stroke volume = (EDV) − (ESV).

Cardiac output = stroke volume × HR.

### 2.10. Statistics

The Shapiro–Wilk statistic was used to test for normality. Differences between two groups were analysed using the unpaired or paired two-tailed *t*-test, as indicated. One-way ANOVA and two-way ANOVA with Holm–Sidak *post hoc* correction for multiple comparisons and multiple *t*-tests were used where indicated. Correlation between two datasets was analysed using linear regression. Datasets of total counts were transformed into log10 values. Data are presented as mean ± SD and *p* < 0.05 was considered statistically significant. * *p* < 0.05, ** *p* < 0.01, *** *p* < 0.001, **** *p* < 0.0001. N indicates the number of embryos or larvae used per dataset. Data were analysed in Graphpad Prism 6 (GraphPad Software, Inc.; La Jolla, CA, USA).

## 3. Results

### 3.1. Tg(myl7:GA) Zebrafish Line Generation

**Figure 1:**
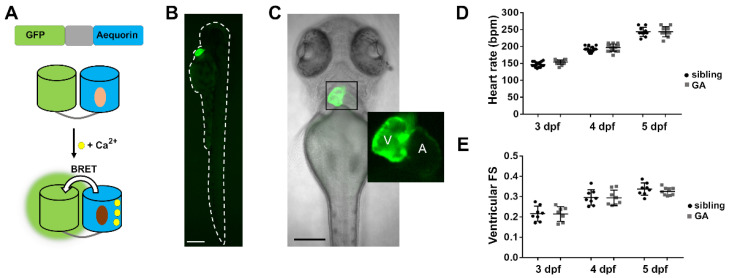
Expression of GA in the heart of zebrafish larvae. (**A**) DNA construct of the chimera GA and scheme of Ca^2+^-dependent bioluminescence. BRET, bioluminescence resonance energy transfer. Light and dark brown ovals represent CTZ and its excited state product. (**B**) GFP fluorescence of a 3 dpf *Tg(myl7:GA)* zebrafish larva. (**C**) Superimposed GFP fluorescence and transmitted light images of a 3 dpf *Tg(myl7:GA)* zebrafish larva. The inset shows the atrium (A) and ventricle (V). (**D**) HR of GA heterozygous and wild-type siblings at 3, 4, and 5 dpf. Data are shown as mean ± SD (*n* = 17 for 3 and 4 dpf; *n* = 10 for 5 dpf). (**E**) Ventricular fractional shortening (FS), measured with the major diameter, of GA heterozygous and sibling larvae at 3, 4, and 5 dpf. Data are shown as mean ± SD (sibling *n* = 8 for 3, 4, and 5 dpf; GA *n* = 7 for 3, *n* = 8 for 4 dpf and *n* = 9 for 5 dpf). Statistical analysis was performed using an unpaired *t*-test in (**D**) and (**E**) and no statistical differences were found between GA-expressing and sibling larvae (*p* > 0.05). The bar scale in (**B**) and (**C**) indicates 250 µm and 150 µm, respectively.

We generated a transgenic zebrafish line expressing GA under the control of the cardiomyocyte-specific promoter *myl7*. Upon Ca^2+^ binding to aequorin, there is energy transfer between the excited state of CTZ and the GFP so that light emission is shifted to the green (Figure 1A) [27,33,38]. The reporter GA is bifunctional: GFP fluorescence is used to determine its localization and expression level, while luminescence reports the Ca^2+^ levels. The expression of GA was observed in the cardiac tube from 24 h post-fertilization and no fluorescence was seen in other organs (Figure 1B). The ventricle was brighter than the atrium at 3, 4, and 5 dpf, due to its thicker wall (Figure 1C). Both the HR and the ventricular fractional shortening (FS) of heterozygous GA larvae were like those observed in wild-type siblings (Figure 1D,E), suggesting that the expression of GA did not affect cardiac function.

### 3.2. Aequorin Reconstitution Protocol

**Figure 2:**
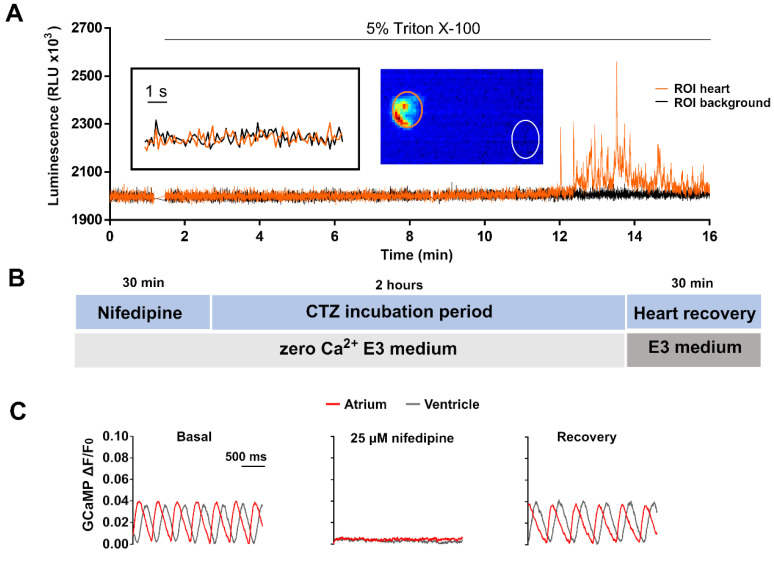
Limiting Ca^2+^ transients in the heart with nifedipine and aequorin reconstitution protocol. (**A**) Representative luminescence of a beating heart in a 3 dpf *Tg(myl7:GA)* larva incubated with 50 µM *diacetyl h*-CTZ in E3 medium for 2 h. Images were acquired at 9 Hz. The image represents the integrated luminescence of the entire experiment, including Triton X-100. Regions of interest (ROI) from heart and background are shown. (**B**) Scheme of the aequorin reconstitution protocol comprising a 30-min incubation with nifedipine in E3_0Ca_ medium to block Ca^2+^ transients, 2 h for aequorin reconstitution with CTZ, and 30 min for recovery of the Ca^2+^ transients in Ca^2+^-containing E3 medium. (**C**) Representative Ca^2+^ transients of a 3 dpf *Tg(myl7:GCaMP)^s878^* larva in basal conditions incubated with 25 μM nifedipine for 30 min in E3_0Ca_ medium, and after the heart recovery period. The fluorescence images were acquired at 200 Hz in a spinning disk confocal microscope; GCaMP fluorescence was measured in a ROI drawn over the ventricle.

Apoaequorin needs to be reconstituted with its substrate CTZ in the presence of oxygen to yield the Ca^2+^-sensitive photoprotein [39]. However, when 3 dpf *Tg(myl7:GA)* zebrafish larvae were incubated in 50 μM *diacetyl h*-CTZ in E3 medium for 2 h, spontaneous Ca^2+^-dependent bioluminescence from the heart was not detected (Figure 2A). Triton-X100 (5%), added to break membranes and bring aequorin into contact with extracellular Ca^2+^, released few counts. We reasoned that, as Ca^2+^ rises during each systole, the rate of aequorin consumption might be faster than the reconstitution rate. Therefore, we hypothesized that limiting Ca^2+^ transients during aequorin reconstitution would improve light output.

#### 3.2.1. Suppressing Ca^2+^ Rise in the Heart with an LTCC Blocker

A protocol was devised to blunt Ca^2+^ transients by incubating larvae with 25 µM nifedipine in nominally zero Ca^2+^ E3 (E3_0Ca_) medium (Figure 2B). Figure S1 shows the optimization of this treatment with different concentrations of nifedipine, with and without Ca^2+^ in the fish water. After 30 min in nifedipine, non-beating larvae were transferred to a plate containing 50 µM CTZ in E3_0Ca_ medium for 2 h (Figure 2B). At the end of this period, the heart remained motionless in 84% of the larvae. Then, larvae were placed in complete E3 medium for 30 min to allow the heartbeat to recover. The Ca^2+^ levels during this protocol were monitored with the Ca^2+^ biosensor GCaMP in the *Tg(myl7:GCaMP)^s878^* zebrafish line (Figure 2C). The Ca^2+^ transients were indeed abrogated by the treatment with nifedipine and restored after the heart recovery period.

#### 3.2.2. Larvae Treated with the Aequorin Reconstitution Protocol Recovered Heart Function after Restoring Ca^2+^ into the Medium

We tested the recovery of cardiac function in *Tg(myl7:GA)* larvae subjected to the aequorin reconstitution protocol. The HR was similar before and after the aequorin reconstitution protocol in 3 dpf (170 ± 9 bpm before vs. 166 ± 15 bpm after; *p* = 0.387), 4 dpf (192 ± 16 bpm before vs. 179 ± 29 bpm after; *p* = 0.163), and 5 dpf larvae (191 ± 16 bpm before vs. 181 ± 24 bpm after; *p* = 0.071) (Figure S2A). In addition, the ventricular FS was restored after the recovery period at 3 dpf (0.23 ± 0.02 for control vs. 0.24 ± 0.02 for Aeq protocol; *p* = 0.832), 4 dpf (0.30 ± 0.04 for control vs. 0.29 ± 0.02 for Aeq protocol; *p* = 0.315), and 5 dpf (0.31 ± 0.02 for control vs. 0.31 ± 0.02 for Aeq protocol; *p* = 0.878) (Figure S2B). Analysis of Ca^2+^ transients in 3 dpf *Tg(myl7:GCaMP)^s878^* larvae indicated that Ca^2+^ transients and their kinetics were similar in the controls and in larvae subjected to the aequorin reconstitution protocol (Figure S2C,E,F). These results suggest that the heart regained normal Ca^2+^ transients, HR and contractility during the recovery period.

#### 3.2.3. Imaging Individual Ventricular Ca^2+^ Transients in the Heart

**Figure 3:**
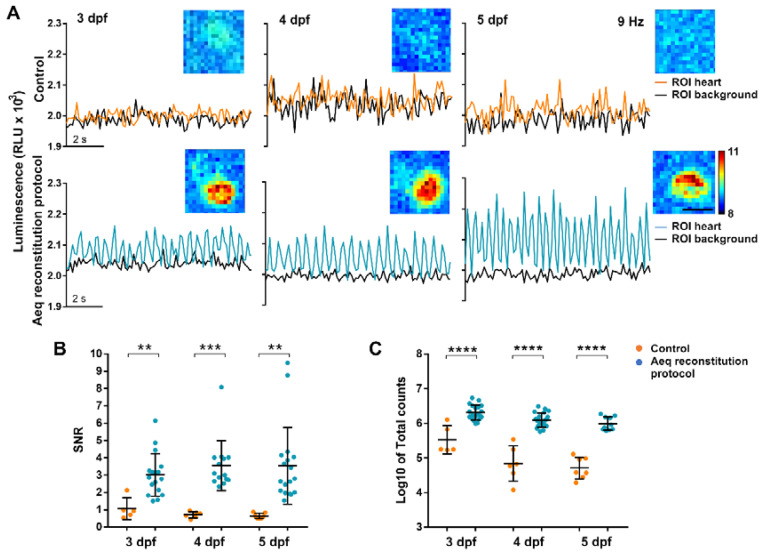
Bioluminescence imaging in the heart of 3, 4, and 5 dpf *Tg(myl7:GA)* zebrafish larvae treated with the aequorin reconstitution protocol. *Diacetyl h*-CTZ was used for reconstitution and images were acquired at 9 Hz. (**A**) Luminescence of CTZ-incubated larvae either with the aequorin reconstitution protocol or in full E3 medium (control). The black lines indicate the luminescence in background ROIs drawn out of the larvae; orange and blue lines show ventricular luminescence. Inset images show the integrated luminescence for 1 min over the heart. The scale bar represents 50 µm and the colour scale indicates RLU. (**B**) SNR of the control (orange) and aequorin reconstitution protocol (blue) larvae at 3, 4, and 5 dpf. Data are shown as mean ± SD (control *n* = 5 for 3 dpf, *n* = 6 for 4 dpf, and *n* = 7 for 5 dpf; aequorin reconstitution protocol *n* = 17 for 3 dpf; *n* = 14 for 4 dpf, and *n* = 18 for 5 dpf). (**C**) Log_10_ of total counts released in the control (orange) and aequorin reconstitution protocol (blue) groups at 3, 4, and 5 dpf. Data are shown as mean ± SD (control *n* = 5 for 3 dpf, *n* = 6 for 4 dpf, and *n* = 7 for 5 dpf; aequorin reconstitution protocol *n* = 19 for 3 dpf; *n* = 21 for 4 dpf, and *n* = 10 for 5 dpf). Statistical analysis was performed using a two-tailed unpaired *t*-test in (b) and (c). ** *p* < 0.01; *** *p* < 0.001 and **** *p* < 0.0001.

We tested the efficiency of the reconstitution protocol by imaging the bioluminescence. Zebrafish *Tg(myl7:GA)* larvae were incubated in 50 µM *diacetyl h*-CTZ for 2 h with or without the previous incubation with 25 µM nifedipine. Cardioluminescence, the spontaneous bioluminescent flashes due to beat-to-beat Ca^2+^ oscillations during the cardiac cycle, was detected only in the ventricle of larvae preincubated with nifedipine (Figure 3A). The signal-to-noise ratio (SNR) of these recordings, a measure of the sensitivity of the method (see Section 2) was higher in larvae treated with nifedipine at 3, 4, and 5 dpf (Figure 3B). Triton X-100 was added to compare the amount of reconstituted aequorin in each case. Larvae preincubated with nifedipine released 5- to 18-fold more counts than the controls (Figure 3C). These results confirmed that limiting the Ca^2+^ transients during the incubation with CTZ increased the amount of functional GA, such that individual ventricular Ca^2+^ transients could be observed. However, spontaneous luminescence from the atrium was not detected in most larvae (Figure S3A–C). Total counts in the atrium after Triton X-100 were 6-fold less than those in the ventricle but were detectable (Figure S3D,E), showing the need to separate spatially the origin of the luminescence. In contrast to photometry, imaging allowed us to draw ROIs over the atrium and ventricle to discriminate their contribution.

Decreasing the image acquisition frequency can improve the SNR of low emitting samples. In fact, a strong inverse correlation was observed between SNR and the acquisition frequency (R^2^ = 0.911; *p* = 0.045) (Figure S4A). In contrast, L/Lmax, which is proportional to Ca^2+^ levels, was independent of the image acquisition frequency (R^2^ = 0.03; *p* = 0.824) (Figure S4B).

### 3.3. Testing Different CTZ Analogues

**Figure 4:**
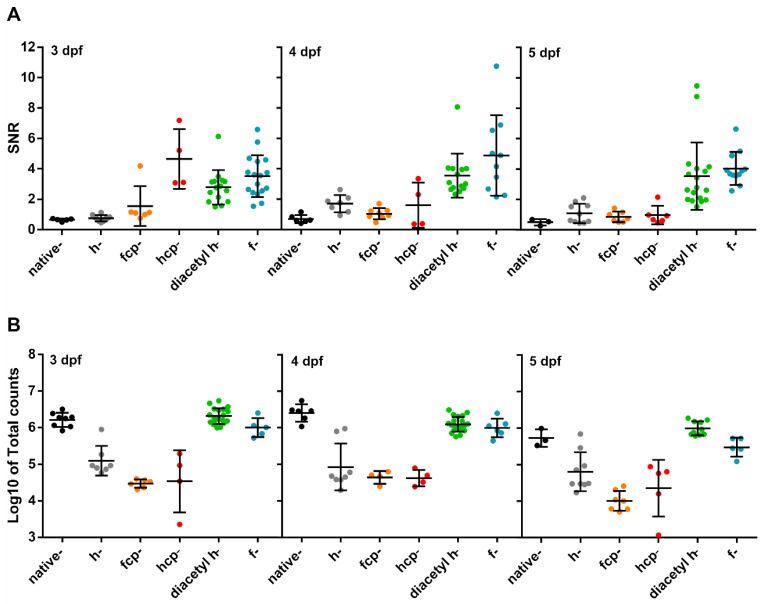
Test of CTZ analogues in 3, 4, and 5 dpf *Tg(myl7:GA)* zebrafish larvae. (**A**) SNR from larvae incubated with the CTZ analogues for 2 h with the aequorin reconstitution protocol. Images were acquired at 9 Hz. (*Native*-CTZ *n* = 5, 6, and 3; *h*-CTZ *n* = 10, 7, and 10; *fcp*-CTZ *n* = 6, 8, and 7; *hcp*-CTZ *n* = 4, 4, and 5; *diacetyl h*-CTZ *n* = 15, 14, and 18; and *f*-CTZ *n* = 17, 10, and 12; for 3, 4, and 5 dpf, respectively). Data are shown as mean ± SD. (**B**) Total counts released by the addition of 5% Triton X-100 in larvae incubated with the CTZ analogues for 2 h. (*Native*-CTZ *n* = 8, 6, and 3; *h*-CTZ *n* = 7, 8, and 9; *fcp*-CTZ *n* = 6, 4, and 7; *hcp*-CTZ *n* = 4, 4, and 5; *diacetyl h*-CTZ *n* = 19, 21, and 10; and *f*-CTZ *n* = 5, 6, and 5; for 3, 4, and 5 dpf, respectively). Data are shown as mean ± SD of Log_10_ of total counts.

Different CTZ synthetic analogues afford varying chemical stability, Ca^2+^ sensitivity and membrane permeability. We, therefore, tested various analogues (Figure S5) to optimize larvae cardioluminescence. The Ca^2+^ sensitivity of *h*-, *f*-, *fcp*-, and *hcp*-CTZ has been shown to be 16-, 20-, 135-, and 190-fold higher than that of *native*-CTZ, respectively [40]. The rate of reconstitution is also affected by the analogue used: *native*-CTZ was 7- to 10-fold faster in cultured cells than *f*- and *h*-CTZ, respectively [41]. Furthermore, *f*-CTZ was reported to have nearly 2-fold more membrane permeability than *native*- and *h*-CTZ [42]. The addition of protective acetyl groups to *h*-CTZ was reported to inhibit autooxidation, improving its chemical stability, and providing a constant supply of substrate [43]. Therefore, we decided to synthesize this analogue to allow long-term imaging. The SNR and the total counts obtained in *Tg(myl7:GA)* larvae from 3 to 5 dpf reconstituted with *native*-, *h*-, *fcp*-, *hcp*-, *f*- and *diacetyl h*-CTZ were evaluated (Figure 4). Both *f*- and *diacetyl h*-CTZ provided the most cardioluminescence, with robust light output at physiological Ca^2+^ levels and high SNR. Therefore, we used *diacetyl h*-CTZ in further experiments.

### 3.4. GA Reports the Effect of Drugs Acting on LTCC in the Zebrafish Ventricle

**Figure 5:**
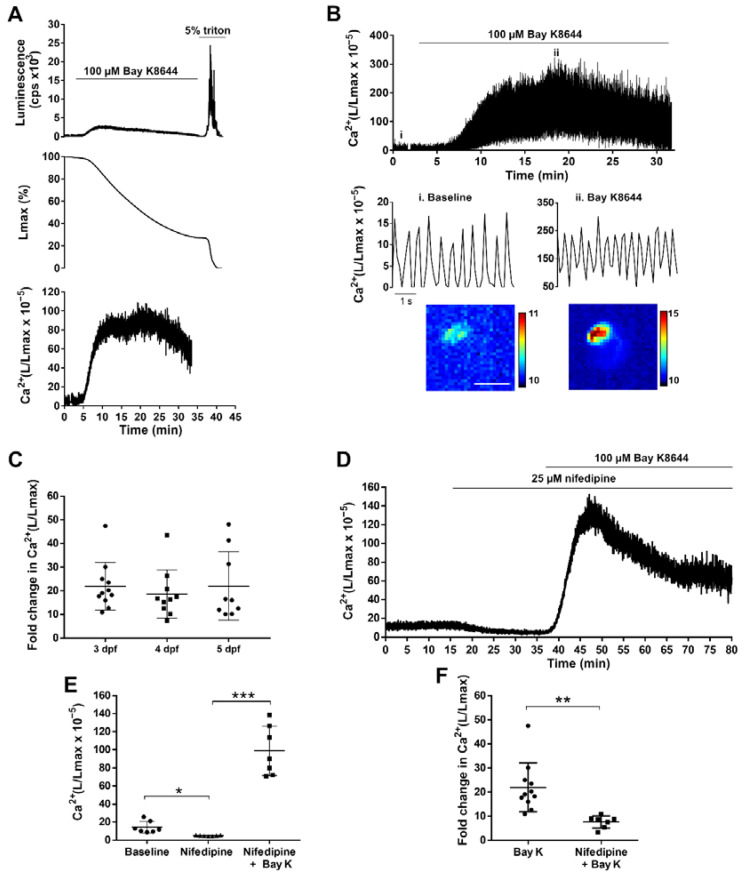
Effect of LTCC activator and antagonist on ventricular Ca^2+^ dynamics of 3, 4, and 5 dpf *Tg(myl7:GA)* zebrafish larvae. (**A**) Analysis of a bioluminescence experiment (3 dpf larva) in which 100 µM Bay K8644 was added from 2 to 33 min, followed by Triton X-100 incubation to release all remaining luminescence counts. Upper panel: Luminescence in counts per s (L, cps); middle panel: remaining counts along the experiment (% of Lmax). Lower panel: L/Lmax, which is proportional to Ca^2+^ levels. Images were acquired at 2 Hz. (**B**) Effect of 100 μM Bay K8644 on the ventricular Ca^2+^ levels. Images were acquired at 9 Hz. The lower panels zoom in the baseline (i) and 100 μM Bay K8644 (ii, min 19). Images of the integrated luminescence during 1 min in baseline and 100 μM Bay K8644 (min 19–20) are shown below. The scale bar represents 100 µm and the colour scale indicates RLU. (**C**) Maximal fold change of the L/Lmax value over basal of larvae treated with 100 μM Bay K8644 at 3, 4, and 5 dpf. Statistical analysis was performed using a one-way ANOVA with Holm–Sidak *post hoc* correction for multiple comparisons and multiple *t*-tests. Data are shown as mean ± SD (*n* = 11 for 3 and 4 dpf, and *n* = 9 for 5 dpf). (**D**) Representative experiment showing the effect of 25 µM nifedipine followed by 100 µM Bay K8644 in a 3 dpf larva. Images were acquired at 1 Hz. (**E**) Maximal effect of 25 µM nifedipine and 100 µM Bay K8644 on ventricular Ca^2+^ levels in 3 dpf larvae treated as in (D). Statistical analysis was performed using a repeated measures one-way ANOVA test. Data are shown as mean ± SD (*n* = 7). (**F**) Fold change in Ca^2+^ (L/Lmax) of 100 µM Bay K 8644 in the absence (*n* = 11) or presence (*n* = 7) of 25 µM nifedipine. Statistical analysis was performed using a two-tailed unpaired *t*-test. Data are shown as mean ± SD. All experiments in this figure were performed with *diacetyl h*-CTZ. * *p* < 0.05, ** *p* < 0.01 and *** *p* < 0.001.

To validate the functionality and sensitivity of the *Tg(myl7:GA)* line, we used two drugs known to increase and decrease Ca^2+^ levels: the LTCC activator Bay K8644 and the LTCC antagonist nifedipine. Figure 5A shows the rationale and analysis of an aequorin experiment in a 3 dpf larva recorded at 2 images/s (2 Hz). This low acquisition frequency was used to track the time-averaged Ca^2+^ levels. The pharmacological approach in zebrafish usually requires higher concentration of drugs than single-cell studies, since drugs must diffuse through the agarose layer, the skin, and tissues to reach the heart. Thus, we used 100 µM Bay K8644 to trigger an increase in luminescence. Then, addition of 5% Triton X-100 caused the release of the remaining aequorin light. It is worth noting that luminescence values have no meaning in terms of Ca^2+^ levels until they are transformed into L/Lmax. This ratio, which is proportional to Ca^2+^ concentration [28], was independent of the total amount of functional aequorin in the sample (Figure S6). To record the effect of Bay K8644 on individual ventricular Ca^2+^ transients, images were acquired at 9 frames/s (9 Hz) (Figure 5B). The Ca^2+^ transient amplitude gradually increased after drug addition, as well as the diastolic and systolic Ca^2+^ levels. Bay K8644 triggered a similar increase in L/Lmax at 3, 4, and 5 dpf (21.9-, 18.7-, and 20.8-fold, respectively) (Figure 5C). 

In contrast with Bay K8644, nifedipine (25 μM) decreased the time-averaged Ca^2+^ levels in 3 dpf larvae imaged at 1 Hz (L/Lmax 14.34 ± 6.49 before nifedipine vs. 4.96 ± 0.50 after nifedipine; *p* = 0.015) (Figure 5D). As images were acquired uninterruptedly, we monitored the reversal of the effect of nifedipine by Bay K8644 (100 μM) on the same larva. The L/Lmax was 4.96 ± 0.50 for nifedipine vs. 98.83 ± 27.33 after Bay K8644 (*p* = 0.0002) (Figure 5D,E). As expected, the increase in L/Lmax induced by Bay K8644 was lower in the presence of nifedipine (7.64-fold ± 2.49 with nifedipine vs. 21.92-fold ± 10.07 without nifedipine; *p* = 0.002) (Figure 5F). Therefore, the *Tg(myl7:GA)* line was sensitive enough to detect both increases and decreases of Ca^2+^ levels, as was shown by modulating Ca^2+^ influx with Bay K8644 and nifedipine. 

Figure S7 compares the effect of Bay K8644 in 3 dpf larvae by cardioluminescence and by fluorescence imaging with GCaMP under similar conditions (heart beating was not stopped). The former allowed continuous recording whereas for GCaMP two periods of imaging (of 5 s duration) were acquired to minimize photobleaching. The GCaMP experiment resolved better the individual Ca^2+^ transients, although it was affected by motion artifacts. A shoulder in the decay phase was observed compared to stopped hearts (*cf.* Figure S7B and Figure 2C). Bioluminescence of GA allowed us to record Ca^2+^ for extended periods; this is a significant advantage as it would be difficult to perform continuous imaging experiments lasting more than 80 min with fluorescent biosensors under widefield microscopy.

### 3.5. Decreasing Adrenergic Tone with Propranolol

**Figure 6:**
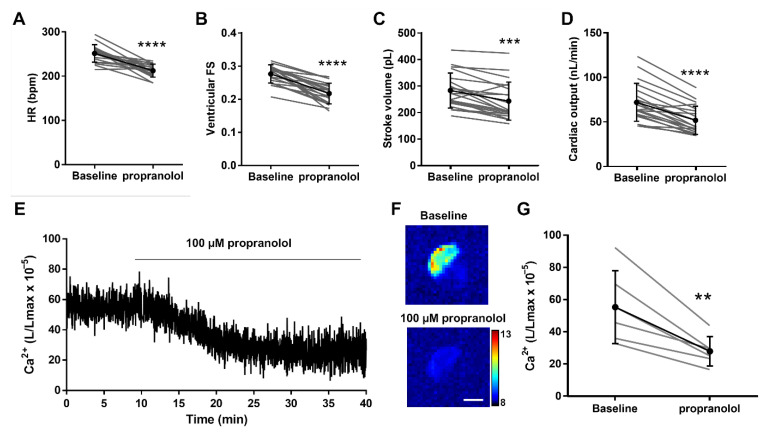
Effect of the β-adrenergic antagonist propranolol on hemodynamic parameters and ventricular Ca^2+^ levels in 4 dpf *Tg(myl7:GA*) zebrafish larvae. Heart rate (HR) (**A**), ventricular fractional shortening (FS) measured with the major diameter (**B**), stroke volume (**C**), and cardiac output (**D**) before (baseline) and after 30 min incubation with 100 µM propranolol. Data are shown as mean ± SD (*n* = 20). (**E**) Representative experiment of the effect of 100 µM propranolol on ventricular Ca^2+^ levels (L/Lmax) (1 Hz image acquisition frequency). (**F**) The images show the integrated luminescence from baseline (min 0–10) and propranolol (min 30–40) periods of the experiment in (**E**). The scale bar represents 100 µm and the colour scale indicates RLU. (**G**) Ca^2+^ levels (L/Lmax) after 30 min of treatment with 100 µM propranolol. Data are shown as mean ± SD (*n* = 6). These experiments were performed using *diacetyl h*-CTZ. Statistical analysis was performed using a two-tailed paired *t*-test. ** *p* <0.01, *** *p* < 0.001 and **** *p* < 0.0001.

We and others reported that the β-adrenergic blocker propranolol decreased systolic and diastolic Ca^2+^ levels in 3 dpf zebrafish larvae using fluorescent biosensors [19,24]. We evaluated the effects of propranolol on cardiac and hemodynamic parameters using the fluorescence of GA in 4 dpf *Tg(myl7:GA)* larvae and tested their Ca^2+^ levels by cardioluminescence. Figure 6A–D and Table 1 shows that the HR, the ventricular FS and fractional area change (FAC), the stroke volume, and the cardiac output decreased after 30 min of incubation with 100 µM propranolol. In agreement with these functional changes, propranolol reduced the Ca^2+^ levels (Figure 6E,F). The L/Lmax decreased 46% (56.8 ± 24.5 baseline vs. 26.1 ± 5.7 after propranolol; *p* = 0.01) (Figure 6G). These results also show that the heart of 4 dpf larvae had some basal adrenergic tone.

### 3.6. Ca^2+^ Levels in a Terfenadine-Induced Heart Failure Model

**Figure 7:**
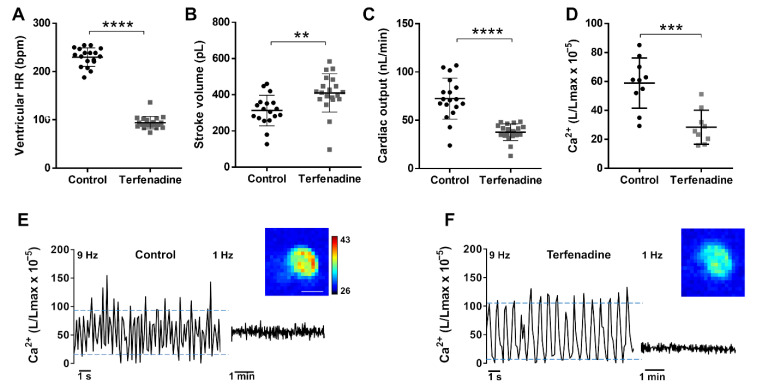
Hemodynamic parameters and Ca^2+^ levels in a terfenadine-induced heart failure model. *Tg(myl7:GA)* larvae (3 dpf) were treated with 10 µM terfenadine for 24 h. Ventricular HR (**A**), stroke volume (**B**), and cardiac output (**C**) of control and terfenadine-treated larvae. Data are shown as mean ± SD (*n* = 18 in control and *n* = 19 in terfenadine). (**D**) Time-averaged Ca^2+^ levels (L/Lmax) of control and terfenadine-treated larvae (1 Hz image acquisition frequency). Data are shown as mean ± SD (*n* = 7 in both groups). Representative experiments of control sibling (**E**) and terfenadine-treated (**F**) larvae showing individual Ca^2+^ transients (9 Hz image acquisition, left part of traces) and the time-averaged levels (1 Hz image acquisition, right part of traces). Upper and lower blue lines indicate the average systolic and diastolic levels. Images of the integrated luminescence during 1 min are shown. The luminescence experiments shown in D, E, and F were performed using *diacetyl h*-CTZ. The scale bar (**E**) represents 100 µm and the colour scale indicates RLU. Statistical analysis was performed using a two-tailed unpaired *t*-test. ** *p* < 0.01; *** *p* < 0.001 and **** *p* < 0.0001.

It has been reported that the treatment of 3 dpf zebrafish larvae with 10 µM terfenadine for 24 h reproduced some features of heart failure, including heart chamber dilatation, reduced FS, arrhythmia, and apoptosis [44,45], but potential changes in Ca^2+^ levels were not assessed. Terfenadine is a potent hERG blocker and can induce prolongation of the QT interval as well as 2:1 atrioventricular block [46]. We measured the hemodynamic parameters and Ca^2+^ levels in this model. The treatment with terfenadine caused bradycardia and a decrease in the atrio-ventricular HR ratio (0.51 ± 0.11) in 94% of the larvae, indicating a 2:1 atrio-ventricular block (Figure 7A). During the missing ventricular excitation, the ventricle remained contracted, suggesting that it was in refractory period due to a prolonged plateau phase and delayed repolarization. The stroke volume was higher in terfenadine-treated larvae than in the controls (Figure 7B). However, since the HR fell markedly, the cardiac output was lower in the terfenadine group than in the controls (Figure 7C). We examined Ca^2+^ levels by cardioluminescence both at high and low image acquisition rate. Terfenadine-treated larvae had lower time-averaged Ca^2+^ levels than the controls; L/Lmax values were 0.48-fold lower in terfenadine larvae (Table 1 and Figure 7D). In contrast, the amplitude of the Ca^2+^ transients increased 2-fold, largely because of increased systolic Ca^2+^ levels (Figure 7E,F and Table 1); this change was in accordance with the enhanced stroke volume.

Both acute incubation with propranolol (Figure 6) and 24 hr treatment with terfenadine (Figure 7) caused decreased cardiac output, but by different mechanisms. In propranolol-treated larvae, the changes observed agree with the expected reduction in adrenergic tone and the measured decrease in average Ca^2+^ levels (0.46-fold decrease) (Table 1). While the end-systolic volume increased 1.29-fold, indicating reduced FAC, the end-diastolic volume did not change, resulting in lower stroke volume (0.86-fold) and ejection fraction (0.86-fold) (Table 1). Since the HR also decreased (0.84-fold, negative chronotropic effect), the cardiac output was 72% of control, and there was no arrhythmia (the atrio-ventricular HR ratio was 1). In the terfenadine model of heart failure, there was an altered contraction pattern, since the FS measured with the major diameter decreased, whereas the FS with the minor diameter increased. Nonetheless, for overall ventricular function the change in ventricular area (and volume) is more meaningful than the FS along the minor or major axis. The FAC increased 1.19-fold. In addition, the end-systolic volume did not change but the end-diastolic volume increased by 1.23-fold, thus, raising the stroke volume (1.31-fold) (Table 1). As the ventricular HR had fallen to 41% of the control value, the cardiac output (stroke volume × HR) dropped to 0.52-fold of control, but the ejection fraction was maintained. While the average Ca^2+^ levels (measured at 1 Hz image acquisition) also decreased (as with propranolol), the amplitude of the Ca^2+^ transients markedly rose (Table 1). Thus, propranolol and terfenadine reduced cardiac output, but the defining changes caused by terfenadine were the 2:1 arrhythmia causing higher end-diastolic volume and enhanced Ca^2+^ amplitude and stroke volume.

## 4. Discussion

Imaging the heart of zebrafish larvae with a HR of about 3–4 Hz (180–240 bpm) requires acquiring images at high speed. In a previous report using widefield fluorescence microscopy with LED illumination at 50 images/s, we obtained excellent SNR and spatial resolution, but recording was limited to short duration periods (~10 s) to avoid photobleaching [24]. Photobleaching was worse when we used laser illumination in scanning or spinning disc confocal microscopy. Thus, when studying the effect of slow-acting drugs, which have to diffuse through the agarose and skin to reach the heart, 10-s imaging periods were acquired every few minutes [24], which made it difficult to detect random arrhythmic events. Light sheet fluorescence microscopy has been shown to be much less harmful than widefield or confocal microscopy, allowing to image cell motion in beating larva hearts, or Ca^2+^ levels in non-contracting hearts [20]. Indeed, Ca^2+^ imaging in the heart with intensiometric fluorescence biosensors requires stopping contraction with drugs or morpholinos against myosin to avoid motion artifacts [19,21]. Another limitation we observed was the autofluorescence of the vitello, which affected mostly the atrium in 3 dpf larvae [24]. The antifungal methylene blue, commonly used in the fish water, and drugs like *para*-amino blebbistatin also increased autofluorescence. To overcome these issues, here we have used bioluminescence imaging of aequorin fused to GFP (GA) to monitor continuously Ca^2+^ levels in experiments of up to 2 h in 3–5 dpf larvae. In these experiments, the heart was performing its mechanical function and the mechano-electrical feedback was preserved.

Reconstitution of apoaequorin with CTZ in single-cells or in vivo is not generally a major problem [26,29,30,32,47]. However, we failed to obtain luminescence in larval hearts with standard protocols and hypothesized that aequorin was rapidly consumed during the reconstitution step as Ca^2+^ is oscillating continuously. It has been previously reported that nifedipine-treated larvae restored the frequency and amplitude of heart Ca^2+^ transients after drug washout [19]. Therefore, we treated larvae with nifedipine in free-Ca^2+^ medium to temporarily abolish Ca^2+^ transients during reconstitution. No deleterious effects on heart function were observed after this aequorin reconstitution procedure (Figure S2). A second possible cause of poor luminescence output was the instability of CTZ in solution. We synthesized *diacetyl h*-CTZ, since the acetyl groups have been shown to protect CTZ from autooxidation and improve luminescence [43]. Thus, an optimized reconstitution protocol with nifedipine incubation to lower Ca^2+^ levels, and using diacetyl h-CTZ as the substrate, resulted in high levels of active aequorin. Notably, *diacetyl h*-CTZ enhanced the SNR and total counts compared to *h*-CTZ (Figure 4), allowing long-term Ca^2+^ imaging in the heart of live larvae. Hence, the diacetyl derivative of *f*-CTZ might further augment cardioluminescence since *f*-CTZ was comparable to *diacetyl h*-CTZ in our results (Figure 4).

As the SNR decreased at high acquisition frequencies (i.e., 25 Hz, Figure S4A), we chose 9 images/s (9 Hz) to maintain a balance between the ability to resolve individual Ca^2+^ transients and the SNR. Lower frame rates (1–2 Hz) increased the SNR and provided information about the time-averaged Ca^2+^ levels, as was shown with both nifedipine and propranolol. This highlights the versatility of GA bioluminescence to monitor oscillatory Ca^2+^ transients or averaged levels.

Incubation of 3 dpf zebrafish larvae with the antihistamine drug terfenadine for 24 h has been reported to induce heart failure [44,45], involving arrhythmia and systolic dysfunction, but potential changes in cytosolic Ca^2+^ were not investigated. The proarrhythmic risk of terfenadine may arise from its numerous effects on cardiomyocyte electrophysiology: block of Na^+^ and L-type Ca^2+^ currents that slows ventricular conduction and promotes non-*Torsades de pointes* ventricular tachycardia and fibrillation [46]; increased frequency of spontaneous Ca^2+^ release from the sarcoplasmic reticulum and enhanced NCX spontaneous currents inducing afterdepolarizations [48]; and block of hERG with QT prolonging effects [46]. In zebrafish, prolonged repolarization results in atrio-ventricular block [49]. Our results indeed showed a 2:1 atrio-ventricular block and decreased ventricular HR (Figure 7 and Table 1). This caused a decrease in average ventricular Ca^2+^ levels but increased Ca^2+^ transient amplitude, resulting in increased stroke volume. The reduction of HR prolongs the diastole, Ca^2+^ extrusion, and reuptake into the sarcoplasmic reticulum, and has been shown to decrease diastolic Ca^2+^ levels [6]. In addition, prolongation of the plateau phase of the action potential by inhibition of zERG would increase the systolic Ca^2+^ levels (Table 1). As a result of the markedly reduced HR, cardiac output decreased. In contrast with terfenadine, the adrenergic block with propranolol caused reduced average Ca^2+^ levels, but a negative inotropic effect (reduced stroke volume). 

Since the bioluminescence reaction of aequorin is triggered by three Ca^2+^ ions [50], the steepness of the Ca^2+^-response curve confers it an excellent SNR. However, in Ca^2+^ microdomains near sarcolemmal Ca^2+^ channels or ryanodine receptors on the sarcoplasmic reticulum surface (Ca^2+^ sparks) [51], a small fraction of total aequorin may be exposed to very high Ca^2+^ concentrations. Thus, L/Lmax may be dominated by these microdomains and may not represent the average cytoplasmic Ca^2+^ levels. In fact, the experiments with Bay K8644, which induced a 20-fold change in L/Lmax, suggest the existence of such microdomains. 

The main limitation of aequorin luminescence methods compared to fluorescence is their low photon yield. The atrium, being thinner than the ventricle, contains fewer cells and thus, less GA. Therefore, we focused on the ventricular Ca^2+^ levels because atrial bioluminescence was under the limit of detection of our imaging system. Nevertheless, atrial bioluminescence was observed when Ca^2+^ levels were increased with Bay K8644. Imaging, in contrast to photometry, allowed us to quantify ventricular Ca^2+^ by setting appropriate ROIs, eliminating any contribution from the atrium. Luminescence may also be close to noise levels when Ca^2+^ levels decrease by drugs (i.e., nifedipine) or in a pathological model, precluding accurate measurements. In the future, the sensitivity issue may be overcome by increasing aequorin’s Ca^2+^ affinity with mutagenesis or with appropriate CTZ analogues [27] and by the continuous development of more sensitive detectors.

In view of the above, the choice of a fluorescent (GCaMP, Twitch-4) or a bioluminescent (GA) biosensor would depend on the research question. Bioluminescence would be preferred when a continuous record of Ca^2+^ is desired (as depicted in Figure S7), for instance, when searching for a paroxysmal arrhythmia. A fluorescence experiment would likely miss these events since short imaging periods of a few seconds every few minutes are normally acquired to avoid photobleaching. In contrast, to see changes on the shape of the transients, like the effect of drugs or mutations prolonging action potential, fluorescence would be better than bioluminescence.

In conclusion, we describe a transgenic model to study Ca^2+^ physiology and pathophysiology in the embryonic zebrafish heart by bioluminescence, as a complementary method to the popular fluorescent Ca^2+^ biosensors. The devised reconstitution protocol increased the amount of functional aequorin without causing deleterious heart effects. Ventricular Ca^2+^ dynamics in beating hearts were imaged continuously for hours in 3–5 dpf larvae. Both the time-averaged Ca^2+^ levels and individual Ca^2+^ transients were monitored and, as a proof-of-concept, we studied changes induced by drugs acting on LTCC and sympathetic input. *Tg(myl7:GA)* larvae also revealed a decrease in time-averaged Ca^2+^ levels, but an increase in Ca^2+^ transient amplitude and stroke volume in a model of heart failure induced by terfenadine. Fluorescence and luminescence imaging have each particular benefits and constraints but are orthogonal techniques able to interrogate different aspects of pathophysiological processes. Cardioluminescence in particular avoids autofluorescence and photobleaching and allows monitoring heart Ca^2+^ for longer periods of time.

## Figures and Tables

**Table 1:** Hemodynamic parameters and Ca^2+^ level values of embryos incubated with 100 µM propranolol (30 min) or 10 µM terfenadine (24 h).

		FS	FS	FAC	EDV	ESV	SV	HR	CO	EF	A/V HR	Aver.Ca	CaT amp	Ca syst	Ca diast
		major diam.	minor diam.		pL	pL	pL/beat	bpm	nL/min			L/Lmax	L/Lmax	L/Lmax	L/Lmax
Control	mean	0.28	0.33	0.39	420.9	137.5	283.3	251	72.0	0.67	1.00	56.8	n.d.	n.d.	n.d.
	SD	0.03	0.06	0.05	89.6	39.7	66.0	20	21.3	0.07	0.00	24.5			
	n	20	20	20	20	20	20	20	20	20	20	6			
Propranolol	mean	0.22	0.27	0.34	420.7	177.2	243.5	212	51.8	0.58	1.00	26.1	n.d.	n.d.	n.d.
	SD	0.03	0.06	0.05	94.7	45.2	71.9	15	15.8	0.08	0.00	5.7			
	n	20	20	20	20	20	20	20	20	20	20	6			
	*p* value	<0.0001	<0.0001	0.001	0.979	<0.0001	0.0001	<0.0001	<0.0001	<0.0001		0.0139			
Fold change	Propranolol/ control	0.79	0.81	0.87	1.00	1.29	0.86	0.84	0.72	0.86	1.00	0.46			
Control	mean	0.36	0.37	0.39	422.1	109.3	312.8	229.9	72.4	0.74	1.00	58.9	61.0	71.6	10.6
	SD	0.04	0.05	0.04	115.3	39.6	84.2	19.1	21.2	0.05	0.00	17.3	28.3	36.0	12.0
	n	18	18	18	18	18	18	18	18	18	18	10	16	16	16
Terfenadine	mean	0.27	0.46	0.47	518.7	108.8	409.9	94.1	37.7	0.78	0.51	28.4	121.5	125.1	4.0
	SD	0.06	0.05	0.08	127.6	31.7	105.7	12.9	8.7	0.06	0.11	11.8	21.4	21.1	2.4
	n	19	19	19	19	19	19	19	19	19	19	9	9	9	9
	*p* value	<0.0001	<0.0001	0.0009	0.019	0.96	0.0037	<0.0001	<0.0001	0.38	<0.0001	0.0004	<0.0001	0.0005	0.12
Fold change	Terfenadine/ control	0.75	1.25	1.19	1.23	1.00	1.31	0.41	0.52	1.06	0.51	0.48	1.99	1.75	0.38

SD, standard deviation; n, number of larvae; FS, fractional shortening; FAC, fractional area change; EDV, end-diastolic volume; ESV, end-systolic volume; SV, stroke volume; HR, heart rate; CO, cardiac output; EF, ejection fraction; A/V HR, atrio-ventricular heart rate ratio; Aver.Ca, average Ca^2+^ level; CaT amp., Ca^2+^ transient amplitude; Ca syst, systolic Ca^2+^ level; Ca diast, diastolic Ca^2+^ level; major diam., ventricle longitudinal diameter; minor diam., ventricle transverse diameter; bpm, beats per minute; n.d., not determined. Statistical analysis was performed using a two-tailed paired *t*-test for propranolol, and a two-tailed unpaired *t*-test for terfenadine.

## Data Availability

The data presented in this study is contained within the article or in Supplementary Material.

## Supplementary Materials

The following are available online at https://www.mdpi.com/article/10.3390/biomedicines9101294/s1, Figure S1: Dose-response of nifedipine on Ca2+ transients measured with GCaMP, Figure S2: Restoration of the heart mechanical function and the Ca^2+^ transients after the recovery period of the aequorin reconstitution protocol, Figure S3: Luminescence in the atrium and the ventricle of 3 dpf *Tg(myl7:GA)* embryos subjected to the aequorin reconstitution protocol, Figure S4: Dependency of SNR and *L/Lmax* on the image acquisition frequency, Figure S5: Structure of the coelenterazines used in this study, Figure S6: Correlation of the basal L/Lmax values versus the total counts in 3 dpf *Tg(myl7:GA)* zebrafish embryos, Figure S7: Comparison of the effect of Bay K8644 (100 µM) in 3 dpf GA expressing embryos and by fluorescence imaging in 3 dpf GCaMP expressing embryos under similar conditions.

## Abbreviations

CTZ: coelenterazine; DMSO, dimethyl sulfoxide; dpf, days post-fertilization; E3_0Ca_ medium, nominally zero Ca^2+^ E3 medium; FAC, fractional area change; FS, fractional shortening; GA, GFP-Aequorin; GFP, green fluorescent protein; HR, heart rate; LTCC, L-type voltage-dependent calcium channel; RLU, relative luminescence unit; ROI, region of interest; SNR, signal-to-noise ratio.
