# Supplementary material for: Cardioluminescence in Transgenic Zebrafish Larvae: A Calcium Imaging Tool to Study Drug Effects and Pathological Modeling
Source: Biomedicines. 2021 Sep 22;9(10):1294. doi: 10.3390/biomedicines9101294 (PMC8533351; doi:10.3390/biomedicines9101294)
Supplement: Supplementary file 1 [file biomedicines-09-01294-s001.zip › biomedicines-1340119-supplementary.pdf]

Cardioluminescence in transgenic zebrafish larvae: a calcium imaging tool to study drug effects and pathological modeling

Manuel Vicente, Jussep Salgado-Almario, Michelle M. Collins, Antonio Martínez-Sielva, Masafumi Minoshima, Kazuya Kikuchi, Beatriz Domingo and Juan Llopis

Supplementary figures:

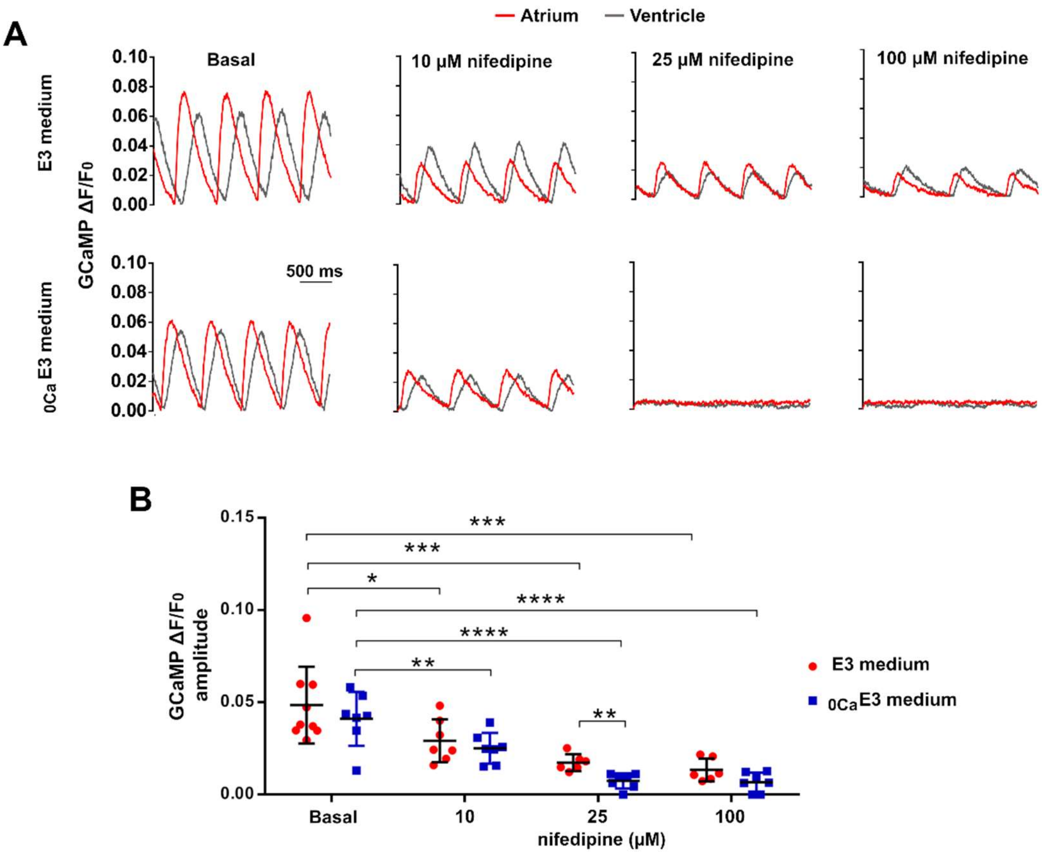

Figure S1. Dose-response of nifedipine on Ca^{2+} transients measured with GCaMP. Three concentrations of nifedipine were tested in the absence or presence of Ca^{2+} in E3 medium in 3 dpf *Tg(myl7:GCaMP)*^{s878} embryos. **(A)** Representative Ca^{2+} transients with 10, 25 and 100 μM nifedipine for 30 min in complete E3 medium (upper panel) or in zero Ca^{2+} E3 ($E3_{0Ca}$) medium (lower panel). The fluorescence images were acquired at 200 Hz. **(B)** Dose-response of nifedipine on ventricular Ca^{2+} transient amplitude in both media. A two-way ANOVA with Holm-Sidak *post hoc* correction for multiple comparisons and multiple *t*-tests was used. Data are shown as the mean ± SD. E3 medium (basal n=9, 10 μM n=7, 25 μM n=6, 100 μM n=6) and $E3_{0Ca}$ medium (basal n=7, 10 μM n=7, 25 μM n=7, 100 μM n=7). * $p < 0.05$, ** $p < 0.01$, *** $p < 0.001$, **** $p < 0.0001$.

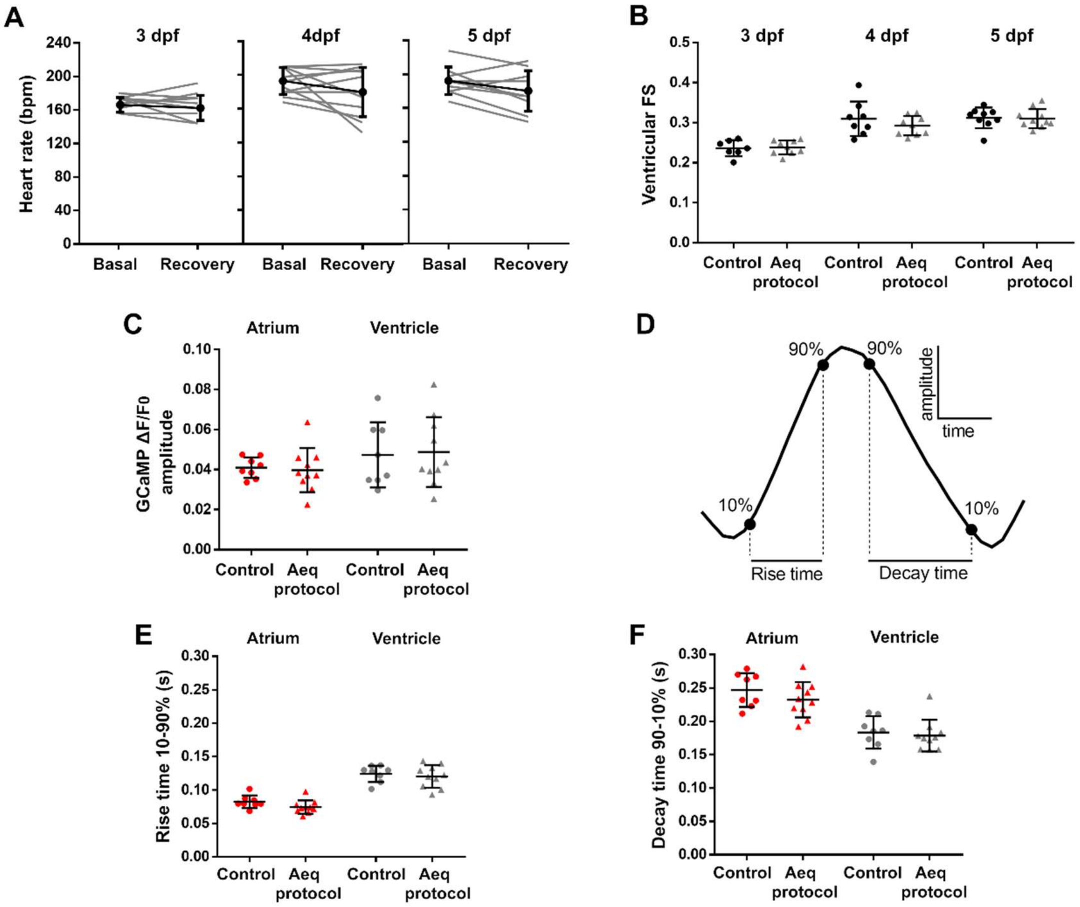

Figure S2. Restoration of the heart mechanical function and the Ca^{2+} transients after the recovery period of the aequorin reconstitution protocol. **(A)** HR during the basal and recovery periods in 3, 4 and 5 dpf *Tg*(*myl7:GA*) zebrafish embryos. A two-tailed paired *t*-test was used. Data are shown as the mean ± SD (n=11 for 3 and 4 dpf; n=10 for 5 dpf). The temperature was held at 27.5ºC. **(B)** Ventricular fractional shortening (FS), measured with the major diameter, from untreated embryos (control group) and the aequorin reconstitution group in 3, 4 and 5 dpf *Tg*(*myl7:GA*) embryos. A two-tailed unpaired *t*-test was used. Data are shown as the mean ± SD (control n=7 for 3 dpf, n=8 for 4 dpf and n=9 for 5 dpf; Aeq protocol n=9 for 3 and 4 dpf and n=10 for 5 dpf). **(C)** Ca^{2+} transient amplitude from control and Aeq reconstitution protocol 3 dpf *Tg*(*myl7:GCaMP*)^{s878} larvae. **(D)** Scheme of a Ca^{2+} transient showing the 10-90% rise time and 90-10% decay time. **(E)** Rise time and **(F)** decay time in atrium and ventricle from untreated embryos (control) and Aeq protocol groups. A two-tailed unpaired *t*-test was used. Data are shown as the mean ± SD (n=8 for control and n=10 for Aeq protocol groups for C, E and F). No statistical differences were found in any of the groups (p > 0.05).

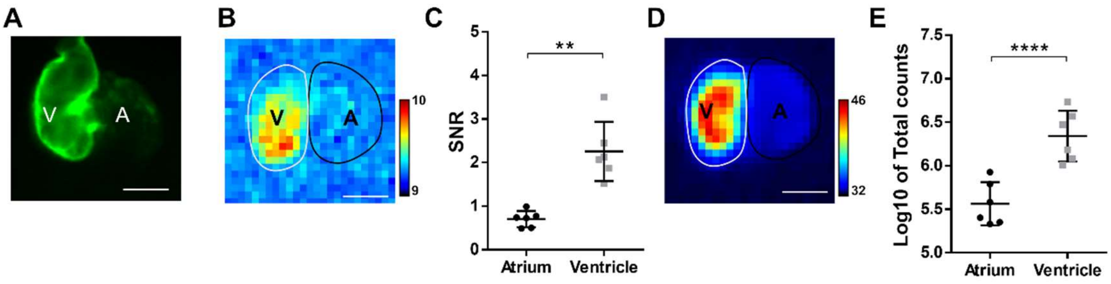

Figure S3. Luminescence in the atrium and the ventricle of 3 dpf *Tg(myl7:GA)* embryos subjected to the aequorin reconstitution protocol. **(A)** Image of GFP fluorescence in the heart showing the atrium (A) and ventricle (V). **(B)** Image of integrated luminescence for 1 min. Images were acquired at 9 Hz. **(C)** SNR of the atrium and the ventricle. A two-tailed paired *t*-test was used. Data are shown as mean ± SD (n=6). **(D)** Image of the luminescence integrated during the whole experiment. Images were acquired at 9 Hz. **(E)** Total counts released in the atrium and in the ventricle. A two-tailed paired *t*-test was used. Data are shown as mean ± SD (n=6). *Diacetyl h*-CTZ was used for reconstitution. Scale bar in (A), (B) and (D) represents 100 μm and the color scale indicates RLU. ** $p < 0.01$, **** $p < 0.0001$.

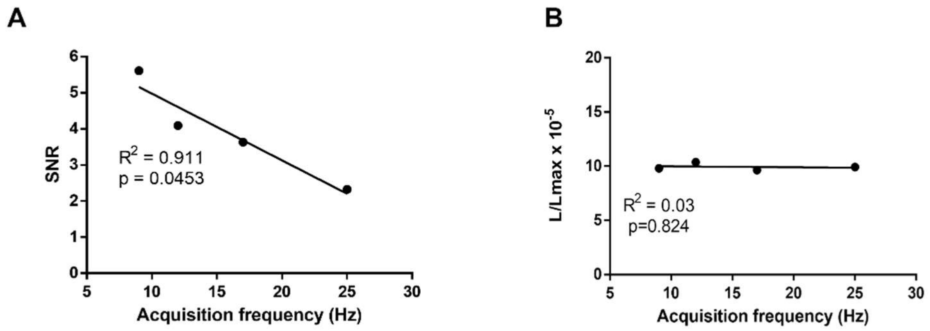

Figure S4. Dependency of SNR and *L/Lmax* on the image acquisition frequency. Correlation between the image acquisition frequency (9, 12, 17 and 25 Hz) and the SNR **(A)** and the *L/Lmax* **(B)** in a representative 3 dpf *Tg(myl7:GA)* zebrafish embryo reconstituted with *diacetyl h*-CTZ.

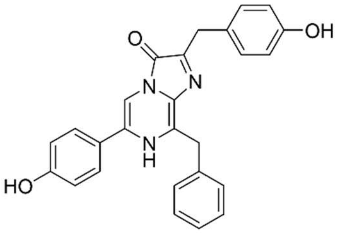

native-CTZ

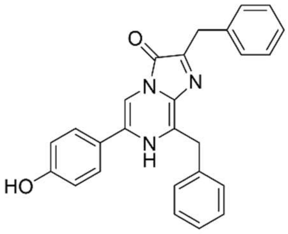

h-CTZ

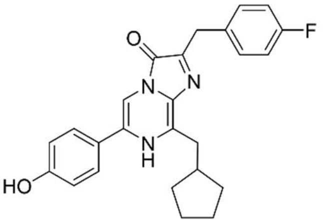

fcp-CTZ

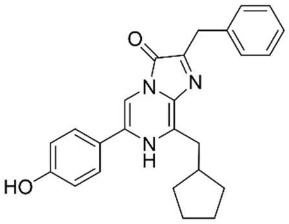

hcp-CTZ

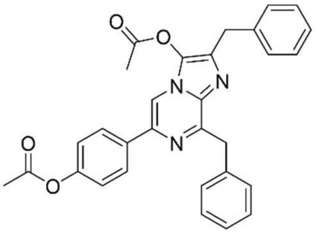

diacetyl h-CTZ

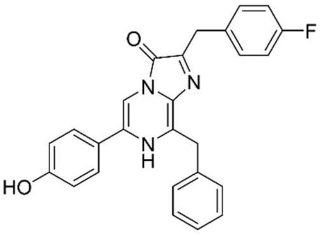

f-CTZ

Figure S5. Structure of the coelenterazines used in this study.

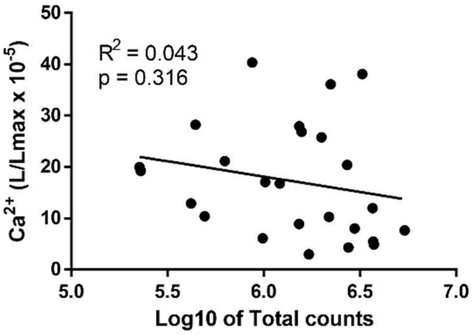

Figure S6. Correlation of the basal L/Lmax values versus the total counts in 3 dpf *Tg(myl7:GA)* zebrafish embryos. These experiments were performed using *diacetyl h*-CTZ. Image acquisition rate was 9 Hz. A linear regression test was used (n=25).

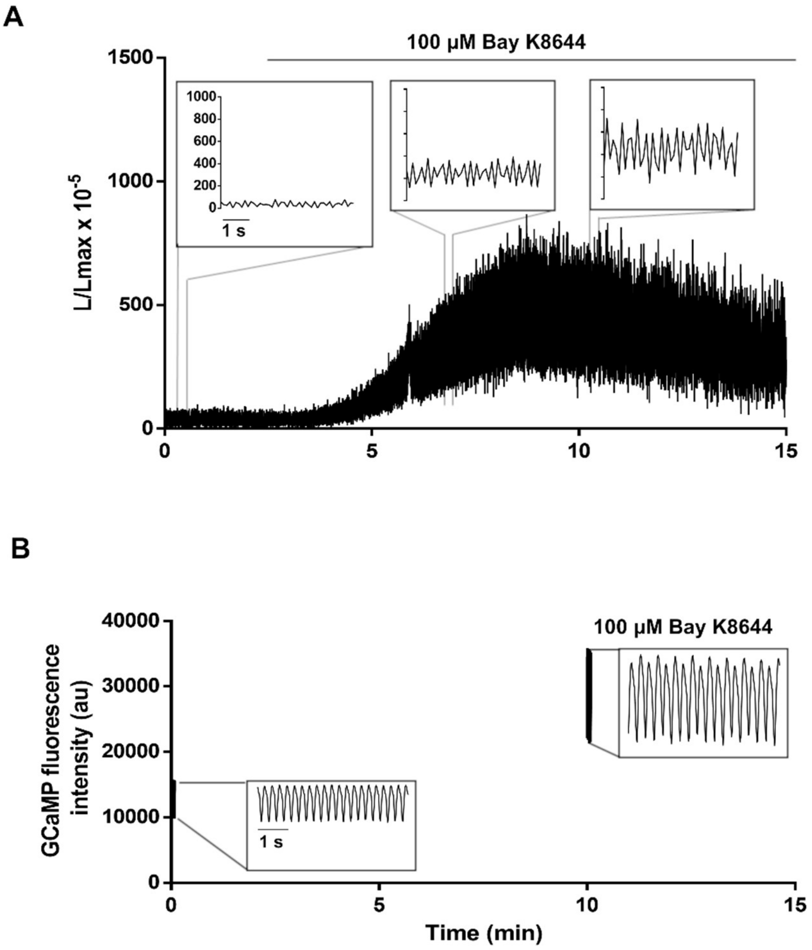

Figure S7. Comparison of the effect of Bay K8644 (100 µM) in 3 dpf GA expressing embryos and by fluorescence imaging in 3 dpf GCaMP expressing embryos under similar conditions (heart beating was not stopped). **(A)** Luminescence in the ventricle after addition of Bay K8644. The insets expand 5 s recording periods. Images were acquired at 9 Hz. *Diacetyl h*-CTZ was used for reconstitution. **(B)** GCaMP fluorescence traces obtained from images acquired during 5 s at the indicated times before and after addition of Bay K8644. The insets expand the time scale to resolve the individual transients.
